# The influence of different signal-to-background ratios on spatial resolution and F18-FDG-PET quantification using point spread function and time-of-flight reconstruction

**DOI:** 10.1186/2197-7364-1-12

**Published:** 2014-09-19

**Authors:** Julian MM Rogasch, Frank Hofheinz, Alexandr Lougovski, Christian Furth, Juri Ruf, Oliver S Großer, Konrad Mohnike, Peter Hass, Mathias Walke, Holger Amthauer, Ingo G Steffen

**Affiliations:** 1 Klinik für Radiologie und Nuklearmedizin, Universitätsklinikum Magdeburg A.ö.R., Otto-von-Guericke Universität Magdeburg, Leipziger Straße 44, Magdeburg, 39120 Germany; 2 PET Center, Institute of Radiopharmaceutical Cancer Research, Helmholtz-Zentrum Dresden-Rossendorf, Landstraße 400, Dresden, 01328 Germany; 3 Klinik für Nuklearmedizin, Universitätsklinikum Freiburg, Hugstetter Straße 55, Freiburg im Breisgau, 79106 Germany; 4 Klinik für Strahlentherapie, Universitätsklinikum Magdeburg A.ö.R., Otto-von-Guericke Universität Magdeburg, Leipziger Straße 44, Magdeburg, 39120 Germany

**Keywords:** FDG-PET/CT reconstruction, PSF, TOF, Spatial resolution, Metabolic tumor volume delineation, Signal-to-background ratio, Radial activity concentration profile, Gibbs artifact, Ringing artifact, Gibbs phenomenon

## Abstract

**Background:**

F18-fluorodeoxyglucose positron-emission tomography (FDG-PET) reconstruction algorithms can have substantial influence on quantitative image data used, e.g., for therapy planning or monitoring in oncology. We analyzed radial activity concentration profiles of differently reconstructed FDG-PET images to determine the influence of varying signal-to-background ratios (SBRs) on the respective spatial resolution, activity concentration distribution, and quantification (standardized uptake value [SUV], metabolic tumor volume [MTV]).

**Methods:**

Measurements were performed on a Siemens Biograph mCT 64 using a cylindrical phantom containing four spheres (diameter, 30 to 70 mm) filled with F18-FDG applying three SBRs (SBR1, 16:1; SBR2, 6:1; SBR3, 2:1). Images were reconstructed employing six algorithms (filtered backprojection [FBP], FBP + time-of-flight analysis [FBP + TOF], 3D-ordered subset expectation maximization [3D-OSEM], 3D-OSEM + TOF, point spread function [PSF], PSF + TOF). Spatial resolution was determined by fitting the convolution of the object geometry with a Gaussian point spread function to radial activity concentration profiles. MTV delineation was performed using fixed thresholds and semiautomatic background-adapted thresholding (ROVER, ABX, Radeberg, Germany).

**Results:**

The pairwise Wilcoxon test revealed significantly higher spatial resolutions for PSF + TOF (up to 4.0 mm) compared to PSF, FBP, FBP + TOF, 3D-OSEM, and 3D-OSEM + TOF at all SBRs (each *P* < 0.05) with the highest differences for SBR1 decreasing to the lowest for SBR3. Edge elevations in radial activity profiles (Gibbs artifacts) were highest for PSF and PSF + TOF declining with decreasing SBR (PSF + TOF largest sphere; SBR1, 6.3%; SBR3, 2.7%). These artifacts induce substantial SUVmax overestimation compared to the reference SUV for PSF algorithms at SBR1 and SBR2 leading to substantial MTV underestimation in threshold-based segmentation. In contrast, both PSF algorithms provided the lowest deviation of SUVmean from reference SUV at SBR1 and SBR2.

**Conclusions:**

At high contrast, the PSF algorithms provided the highest spatial resolution and lowest SUVmean deviation from the reference SUV. In contrast, both algorithms showed the highest deviations in SUVmax and threshold-based MTV definition. At low contrast, all investigated reconstruction algorithms performed approximately equally. The use of PSF algorithms for quantitative PET data, e.g., for target volume definition or in serial PET studies, should be performed with caution - especially if comparing SUV of lesions with high and low contrasts.

**Electronic supplementary material:**

The online version of this article (doi:10.1186/2197-7364-1-12) contains supplementary material, which is available to authorized users.

## Background

Combined positron-emission tomography/computed tomography (PET/CT) - primarily using F18-fluorodeoxyglucose (FDG) to visualize focal glucose hypermetabolism as an indicator of neoplastic tissue - has proven its significant impact on the therapeutic management in several tumor entities, e.g., non-small cell lung cancer, colorectal cancer, or breast cancer, when compared to conventional imaging methods [1]–[3].

Furthermore, quantitative analyses of FDG-PET findings, mainly expressed as standardized uptake values (SUVs), metabolic tumor volume (MTV), or total lesion glycolysis (TLG), can be helpful for outcome prediction or therapy response assessment [4, 5]. Additionally, with regard to planning procedures for radiotherapy, the use of FDG-PET for target volume definition may enable dose escalation, a lower exposure of organs at risk, as well as reduced interobserver variability [6, 7].

The reconstruction algorithm used for image generation can have substantial influence on quantitative data [8, 9]. Recent reconstruction algorithms commercially available for clinical purposes encompass iterative calculations, time-of-flight (TOF) analysis (to approximate the real location of the positron-electron annihilation), and the point spread functions (PSF) of the PET scanner to account for its specific detection properties. Recent studies revealed systematically higher SUV and smaller MTV when applying such algorithms compared to ordered subset expectation maximization (OSEM) algorithms [10–13]. In contrast, enhanced spatial resolution as well as higher signal-to-noise ratios (SNR) can lead to improved image quality and lesion detection [14]–[16].

The aim of the present study was to investigate the effects of PSF and TOF integration at different SBRs as they typically occur in clinical FDG-PET measurements.

## Methods

### Phantom

A cylindrical phantom (diameter, 20 cm; volume, 6,595 ml) containing four spheres was used. All spheres (diameter 1, 29.9 mm; diameter 2, 39.8 mm; diameter 3, 49.9 mm; diameter 4, 69.7 mm) were initially (measurement 1) filled with a solution of F18-FDG with an activity concentration of 36.8 kBq/ml. The background volume featured an initial activity concentration of 2.3 kBq/ml resulting in a signal-to-background ratio (SBR) of 16.2:1 (SBR1). To examine the influence of different SBRs, further F18 activity was subsequently added to the background before the scanning process was repeated twice (SBR2, 6.0:1; SBR3, 2.3:1). Please see Table 1 for details.

**Table 1:** **Activity concentrations present at each measurement**

Measurement	Administered activity in MBq	Activity concentration (spheres) in kBq/ml	Activity concentration (background) in kBq/ml	SBR
1	34.2	36.8	2.3	16.2
2	53.6	28.9	4.8	6.0
3	124.8	20.9	9.0	2.3

Decay-corrected administered activities (spheres + background), activity concentrations within the spheres and the surrounding background, as well as the respective SBR displayed for each measurement.

### FDG-PET/CT scanning

FDG-PET/CT imaging was performed using a dedicated PET/CT device with an enhanced axial bed coverage of 216 mm (TrueV®) and a 64-slice CT component (Biograph mCT 64®, Siemens Healthcare, Erlangen, Germany). The phantom was positioned in the center of the field of view and measured over two bed positions covering a distance of 345 mm (overlap, 87 mm) with a scan time of 3 min/bed position. CT data were acquired for attenuation correction (X-ray tube current, 50 mA; voltage, 120 kV; 0.5 s/rotation; pitch factor, 0.8).

### Image reconstruction

FDG-PET raw data were reconstructed with six algorithms and respective presets provided by the manufacturer: filtered backprojection (FBP), FBP + time-of-flight analysis (FBP + TOF), 3D-OSEM (iterations, 2; subsets, 24), 3D-OSEM + TOF (iterations, 2; subsets, 21), iterative reconstruction with system-specific PSF modeling (TrueX®, ‘HD∙PET’; iterations, 2; subsets, 24), and PSF + TOF (‘ultraHD∙PET’; iterations, 2; subsets, 21) [15]. The projection data were reconstructed into 200 × 200 × 70 matrices (slice thickness, 5 mm) and into 200 × 200 × 116 matrices (slice thickness, 3 mm). In-plane voxel size was always 4.1 × 4.1 mm. After reconstruction, a Gaussian filter (full width at half maximum [FWHM], 2 mm) was applied. Attenuation correction CT raw data were reconstructed with a slice thickness of 3 and 5 mm with a special filter for low-dose CT (B19f Low Dose ECT).

### Spatial resolution/Gibbs artifacts

The spatial resolution was assessed as the FWHM of the point spread function in the reconstructed images which was modeled by a 3D Gaussian. FWHM was determined by applying the method described in detail by Hofheinz et al. [17]. This method is based on fitting the analytic solution for the radial activity profile of a homogeneous sphere convolved with a 3D Gaussian to the reconstructed data. In this process, the full 3D vicinity of each sphere is evaluated by transforming the data to spherical coordinates relative to the respective sphere's center. The analytic solution has five parameters: signal (true activity within the sphere), background level, FWHM of the PSF, and the radius as well as the (cold) wall thickness of the spherical inserts. The wall thickness was fixed to its known value (1.2 mm). The remaining four parameters were determined by non-linear least squares fits. This method assumes that locally (over a distance of approximately the diameter of the spheres) the PSF is homogeneous and that there is no notable difference between axial and transaxial resolution. Since the spheres were located close to the radial center of the field of view, this assumption is justifiable (see discussion in [17]).

The same profiles were used to determine the magnitude of the *Gibbs* artifacts as described in [18]. For this, a smoothing spline [19] was fitted to the data. The local minimum and maximum (*A*^-^ and *A*^+^, respectively) of the spline were determined. The magnitude of the Gibbs artifacts GA is then given by

1 $$\text{GA}\;=\;\frac{A^{+}-A^{-}}{A^{+}+A^{-}}$$

**Figure 1:**
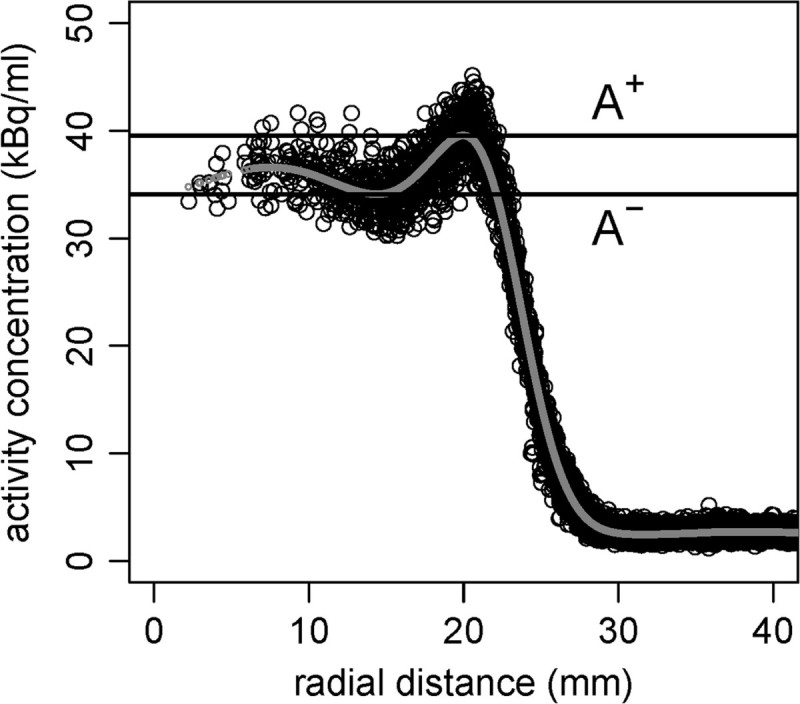
**Determination of Gibbs artifacts (GA).** The black circles represent the radial profile and the gray line depicts the smoothing spline. The black horizontal lines show *A*^+^ and *A*^-^ determined from the smoothing spline.

The determination of GA is illustrated in Figure 1. Obviously, the computation of GA requires a sphere diameter which is large enough that, in principle, a local minimum inside the sphere can occur. Otherwise, the minimum on one side of the sphere overlaps with the Gibbs artifacts of the opposite side, leading to an underestimated GA. Therefore, GA was only determined for the two largest spheres (50 and 70 mm).

### Reference SUV and reference volumes

The reference SUV within the spheres was calculated according to

2 $$\text{SUV}=\frac{\text{Activity}\;\text{concentration}\;\left(\text{kBq}/\text{ml}\right)}{\text{Administered}\;\text{activity}\;\left(\text{MBq}\right)/\text{weight}\;\left(\text{kg}\right)}$$

Based on decay-corrected F18-FDG activities according to the phantom filling protocol, the resulting reference SUVs were 7.1 (SBR1), 3.6 (SBR2), and 1.1 (SBR3). The reference volume for each sphere corresponds to its known physical volume (volume 1, 13.6 ml; volume 2, 33.3 ml; volume 3, 64.7 ml; volume 4, 176.8 ml).

### Volume segmentation

Based on the reconstructed images, sphere volumes were delineated using dedicated software (ROVER, version 2.1.4, ABX advanced biochemical compounds GmbH, Radeberg, Germany). Segmentation was performed for each reconstruction algorithm and the three SBRs, respectively, with the use of four segmentation methods (*t40*, *t50*, *t60*, *tBC*). t40, t50, and t60 (fixed threshold) delineate all voxels with an activity concentration of at least 40%, 50%, or 60% of the measured maximum activity concentration, respectively. The automatic, background-corrected thresholding method (tBC) takes as input a user-defined initial delineation. We used a fixed threshold of 50% of the maximum for this purpose. Then the algorithm iteratively determines the local background of the target structure. After determination, the background is subtracted and a threshold of 39% of the maximum is applied. The delineation is independent of the initial delineation as long as the initial threshold is above the background level (see [20] for details). For all delineations, absolute and relative deviations from the reference volume were calculated.

### Statistical analysis

Data analyses were carried out using R 2.15.3 (Foundation for Statistical Computing, Vienna, Austria, 2012, http://www.R-project.org). Descriptive values are given as mean and range. Signed relative differences were used for comparison of measured quantitative data and their respective reference values. Multivariate general linear models (GLM) including reconstruction algorithms, sphere diameter, SBR, and slice thickness of the reconstructed PET data were used to analyze the association between these factors. Differences of spatial resolution between reconstruction algorithms were investigated using the Friedman test and Wilcoxon test for paired non-parametric data. The one-sample *t* test was performed to detect deviations from reference values. A *P* value of <0.05 was considered as statistically significant.

## Results

### Spatial resolution

**Figure 2:**
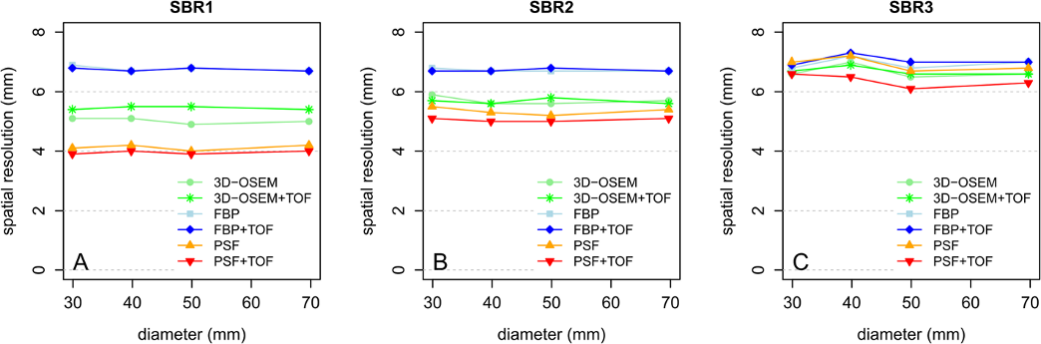
**Spatial resolution displayed as a function of reconstruction algorithm, sphere diameter, and SBR. (A)** SBR1. **(B)** SBR2. **(C)** SBR3.

The spatial resolution of iteratively reconstructed images declined with lower SBR while FBP and FBP + TOF provided relatively constant values (Figure 2). The highest mean resolution at SBR1 as well as SBR2 was provided by PSF + TOF, followed by PSF, 3D-OSEM/3D-OSEM + TOF, and FBP/FBP + TOF. SBR3 showed the smallest differences between mean spatial resolutions of all reconstruction algorithms. Please see Table 2 for details.

**Table 2:** **Spatial resolution and magnitude of Gibbs artifacts (GA)**

	Spatial resolution (mm)	GA (%; diameter, 50/70 mm)
**SBR1**		
FBP	6.8 (6.7 to 6.9)	0.0/0.4
FBP + TOF	6.8 (6.7 to 6.8)	0.0/0.1
3D-OSEM	5.0 (4.9 to 5.1)	0.7/0.5
3D-OSEM + TOF	5.5 (5.4 to 5.5)	0.7/0.7
PSF	4.1 (4.0 to 4.2)	7.3/6.3
PSF + TOF	4.0 (3.9 to 4.0)	7.4/6.3
**SBR2**		
FBP	6.7 (6.7 to 6.8)	0.1/0.3
FBP + TOF	6.7 (6.7 to 6.8)	0.0/0.2
3D-OSEM	5.7 (5.6 to 5.9)	1.4/1.2
3D-OSEM + TOF	5.7 (5.6 to 5.8)	0.7/1.2
PSF	5.3 (5.2 to 5.5)	5.0/4.6
PSF + TOF	5.0 (5.0 to 5.1)	5.1/5.0
**SBR3**		
FBP	7.0 (6.8 to 7.2)	0.1/0.2
FBP + TOF	7.1 (6.9 to 7.3)	0.0/0.0
3D-OSEM	6.7 (6.5 to 7.0)	0.2/1.2
3D-OSEM + TOF	6.7 (6.6 to 6.9)	0.5/1.2
PSF	6.9 (6.7 to 7.2)	1.6/2.6
PSF + TOF	6.4 (6.1 to 6.6)	2.6/2.7

Mean (range) spatial resolutions of all spheres and GA of the two largest spheres are displayed for each SBR and reconstruction method based on 3-mm slice thickness.

Joint analysis of resolution data derived from PET data with 3- and 5-mm slice thickness showed significant differences between reconstruction methods (Friedman rank sum test, *P* < 0.001). The pairwise Wilcoxon test revealed significantly higher mean spatial resolutions for PSF + TOF compared to FBP, FBP + TOF, 3D-OSEM, and 3D-OSEM + TOF at all SBRs (each *P* < 0.05). Similarly, PSF provided significantly higher mean values at SBR1 and SBR2 compared to FBP-based and 3D-OSEM-based reconstructions (each *P* < 0.05) while providing a lower mean spatial resolution at SBR3 compared to 3D-OSEM/3D-OSEM + TOF (each *P* < 0.05) but not compared to FBP/FBP + TOF. PSF + TOF provided significantly higher mean spatial resolutions compared to PSF for all SBRs (SBR1, 4.0 vs. 4.1 mm; SBR2, 5.0 vs. 5.3 mm; SBR3, 6.4 vs. 6.9 mm; each *P* < 0.05). Comparing 3- to 5-mm slice thickness, the spatial resolution improved significantly (each *P* < 0.01) for all reconstruction methods with mean relative changes ranging between 1.1% (PSF; range, 0.0 to 1.5%) and 7.0% (PSF + TOF; range, 5.1 to 7.7%).

### Gibbs artifacts

**Figure 3:**
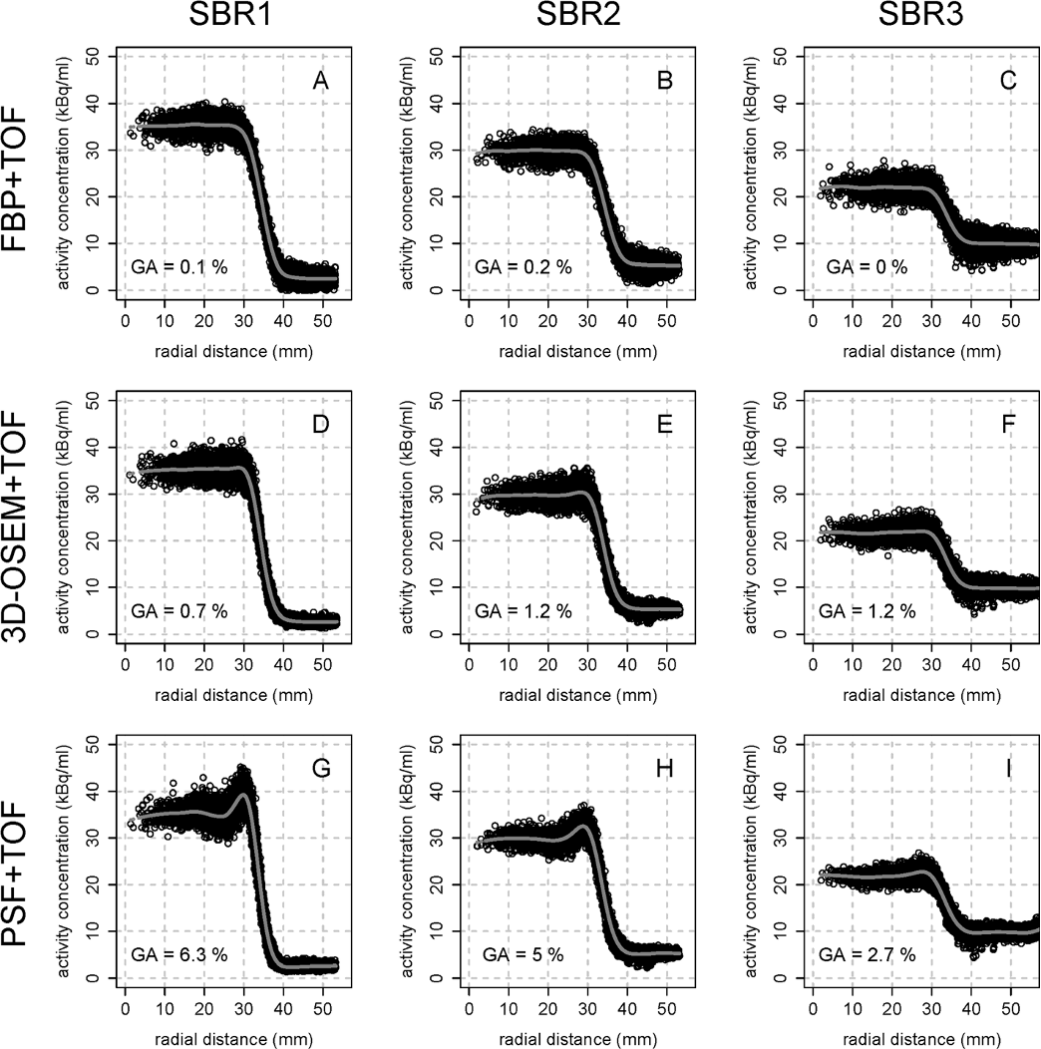
**Radial activity profiles of the largest sphere (70 mm) depending on reconstruction algorithm and SBR.** Edge elevations (Gibbs artifacts) can be observed after reconstruction with PSF + TOF and PSF (not displayed) at SBR1 **(A, D, G)** and SBR2 **(B, E, H)**. SBR3 **(C, F, I)** shows no considerable artifacts. The gray lines indicate the respective smoothing spline.

**Figure 4:**
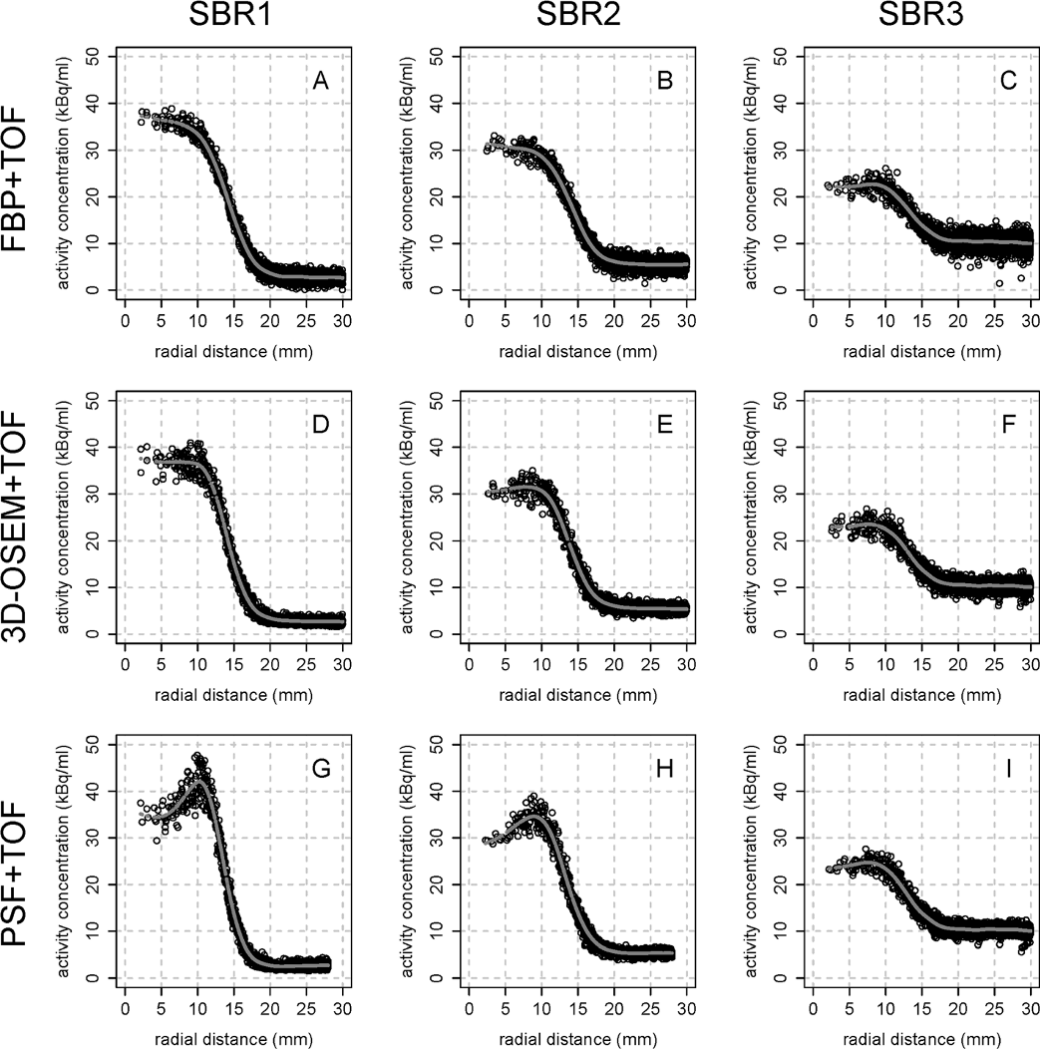
**Radial activity profiles of the smallest sphere (30 mm) depending on reconstruction algorithm and SBR.** Edge elevations (Gibbs artifacts) can be observed after reconstruction with PSF + TOF and PSF (not displayed) at SBR1 **(A, D, G)** and SBR2 **(B, E, H)**. SBR3 **(C, F, I)** shows no considerable artifacts. The gray lines indicate the respective smoothing spline.

Figures 3 and 4 show the radial activity concentration profiles of the largest sphere (70 mm) and smallest sphere (30 mm), respectively, depending on the reconstruction algorithm (FBP + TOF vs. 3D-OSEM + TOF vs. PSF + TOF) and SBR. Each profile displays the activity concentration distribution from the center of the sphere to the surrounding background. The gray line indicates the respective smoothing spline. Notable Gibbs artifacts are visible only for PSF + TOF and PSF (not displayed) at contrasts SBR1 and SBR2 independent of the spheres' diameter which is confirmed by quantification (GA) for the spheres with a diameter of 70 and 50 mm (Table 2). At SBR3, the Gibbs artifacts of both PSF algorithms are clearly reduced. FBP- and 3D-OSEM-based reconstructions showed no notable artifacts at all contrasts and diameters.

### SUVmax

**Figure 5:**
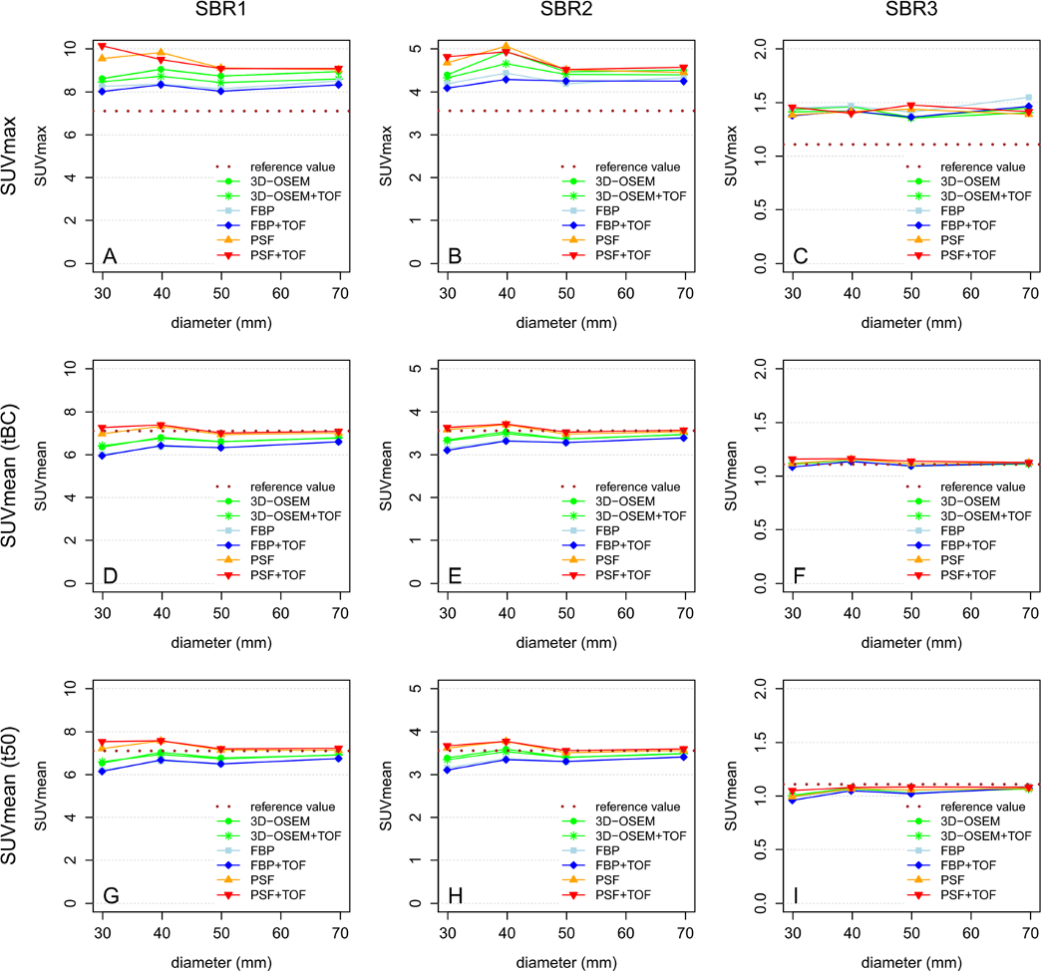
**SUVmax/SUVmean displayed as a function of reconstruction algorithm, sphere diameter, and SBR.** SUVmean based on segmentation with tBC or with t50, respectively. **(A, D, G)** SBR1. **(B, E, H)** SBR2. **(C, F, I)** SBR3.

Comparing the SUVmax with the reference SUV, the one-sample *t* test showed significant differences for all reconstruction methods at all SBRs (Figure 5A,B,C; each *P* < 0.01). Both PSF algorithms resulted in the highest mean relative deviations at SBR1 and SBR2 compared to 3D-OSEM, 3D-OSEM + TOF, FBP, and FBP + TOF. At SBR3, all reconstruction algorithms provided comparable values for SUVmax (see Table 3 for details).

**Table 3:** **Deviations of SUVmax and SUVmean from reference SUV**

	ΔSUVmax (%)	ΔSUVmean (%) [tBC]	ΔSUVmean (%) [t50]
**SBR1**			
FBP	17.0 (14.7 to 19.5)***	-11.0 (-15.4 to -7.3)**	-8.2 (-12.7 to -5.0)*
FBP + TOF	15.1 (12.8 to 17.2)**	-11.0 (-16.3 to -7.1)*	-8.3 (-13.5 to -5.0)*
3D-OSEM	24.2 (21.1 to 27.2)***	-6.6 (-10.6 to -4.2)*	-4.1 (-8.1 to -0.9)
3D-OSEM + TOF	20.2 (18.5 to 22.6)***	-6.6 (-9.7 to -4.5)*	-4.3 (-7.2 to -2.3)*
PSF	31.9 (26.9 to 38.2)**	-0.6 (-2.4 to 3.1)	2.3 (0.5 to 6.6)
PSF + TOF	32.9 (27.7 to 42.7)**	1.2 (-1.3 to 4.0)	3.9 (1.4 to 6.7)
**SBR2**			
FBP	20.4 (17.6 to 24.5)**	-7.6 (-11.7 to -4.4)*	-6.9 (-11.4 to -3.9)*
FBP + TOF	18.5 (14.9 to 20.3)***	-8.0 (-12.8 to -4.8)*	-7.5 (-12.7 to -4.2)*
3D-OSEM	28.5 (23.4 to 38.8)**	-3.6 (-6.0 to -0.7)	-2.5 (-4.7 to 1.0)
3D-OSEM + TOF	24.8 (21.4 to 30.7)**	-4.1 (-6.6 to -2.0)*	-3.3 (-5.9 to -0.6)
PSF	31.3 (24.9 to 42.3)**	0.5 (-2.2 to 4.0)	1.7 (-1.3 to 6.1)
PSF + TOF	32.3 (26.9 to 38.5)**	1.5 (-0.9 to 4.4)	2.6 (0.1 to 6.1)
**SBR3**			
FBP	32.4 (27.1 to 39.6)**	1.4 (-1.1 to 3.5)	-6.1 (-10.9 to -1.4)
FBP + TOF	26.9 (23.0 to 32.1)***	-0.1 (-2.3 to 2.3)	-7.5 (-13.5 to -3.2)*
3D-OSEM	28.7 (22.9 to 31.8)***	0.7 (-1.5 to 3.4)	-5.8 (-9.3 to -3.2)*
3D-OSEM + TOF	26.1 (22.2 to 28.1)***	0.8 (-0.9 to 2.9)	-6.2 (-9.6 to -4.0)*
PSF	26.9 (24.8 to 29.6)***	1.8 (0.6 to 4.4)	-5.6 (-10.5 to -3.4)*
PSF + TOF	29.6 (26.2 to 33.2)***	3.3 (1.5 to 4.8)*	-3.2 (-5.3 to -2.3)*

Mean (range) relative deviations from reference SUV of all spheres are displayed for each SBR and reconstruction method. *t* test: *P < 0.05; **P < 0.01; ***P < 0.001.

The SUVmax was significantly associated with reconstruction algorithm (reference method, 3D-OSEM; each *P* < 0.01), sphere diameter (*P* < 0.001), SBR (reference SBR, SBR1; each *P* < 0.001), and slice thickness of the reconstructed PET data (*P* < 0.001) in GLM.

### SUVmean

Compared to SUVmax, the measured SUVmean after semiautomatic segmentation (tBC) showed a higher agreement with the reference SUV (Figure 5D,E,F). In contrast to the former, both PSF algorithms provided smaller mean relative deviations of the SUVmean from the reference SUV at SBR1 as well as SBR2 compared to 3D-OSEM, 3D-OSEM + TOF, FBP, and FBP + TOF. Again, smaller differences were observed at SBR3 between all reconstruction algorithms investigated. Please see Table 3 for details. The SUVmean resulting from segmentation with a fixed threshold (t50) is displayed in Figure 5G,H,I for comparison.

In GLM, the SUVmean was significantly associated with reconstruction algorithm (reference method, 3D-OSEM; FBP, *P* < 0.05; FBP + TOF, *P* < 0.05; 3D-OSEM + TOF, *P* = 0.7; PSF, *P* < 0.001; PSF + TOF, *P* < 0.001), sphere diameter (*P* < 0.05), and SBR (reference SBR, SBR1; each *P* < 0.001) but not with the slice thickness of the reconstructed PET data (*P* = 0.17).

### MTV deviation from reference volumes

**Figure 6:**
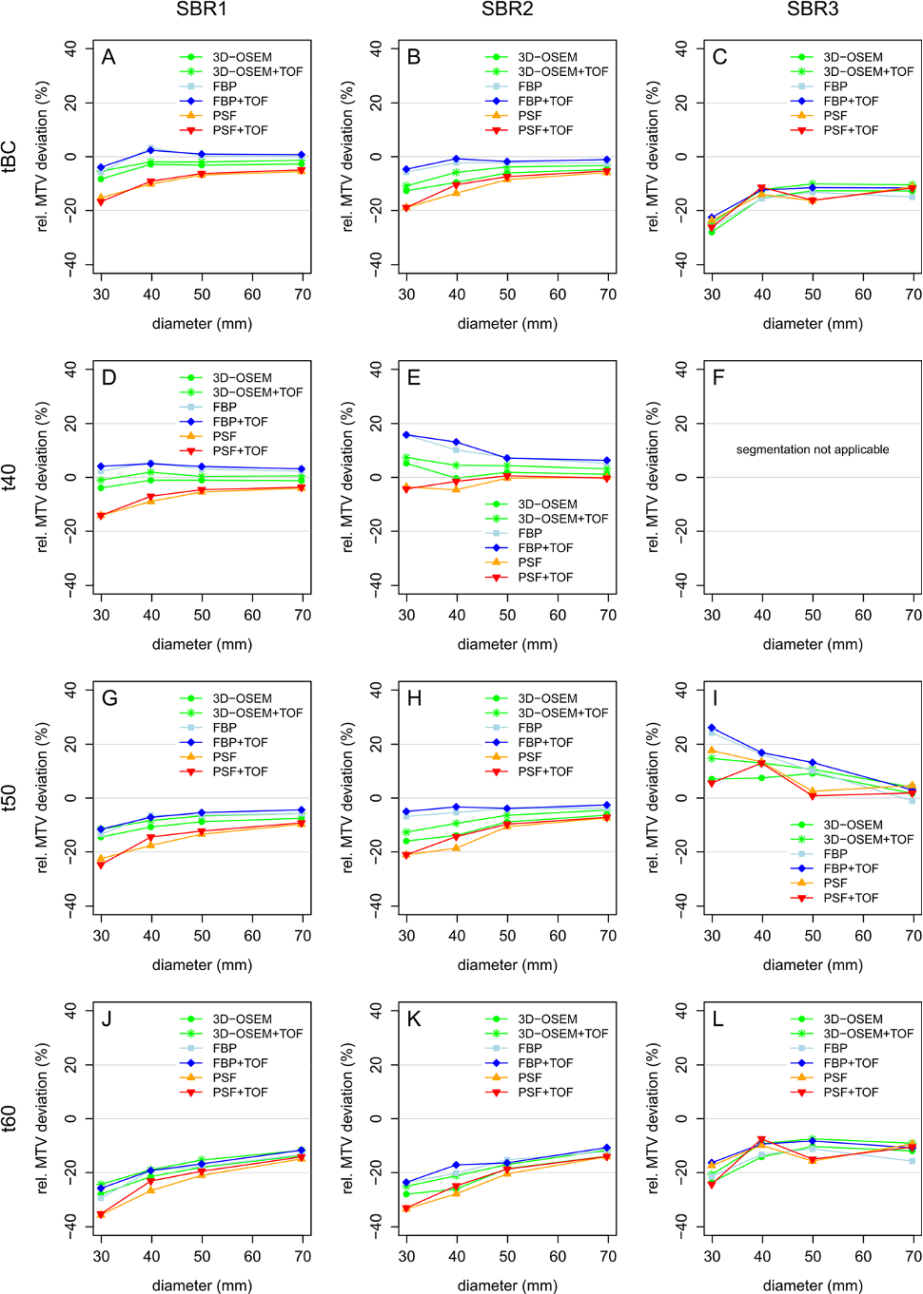
**Relative MTV deviations displayed as a function of reconstruction algorithm, sphere diameter, and SBR. (A, D, G, J)** SBR1. **(B, E, H, K)** SBR2. **(C, F, I, L)** SBR3.

Figure 6 displays the relative MTV deviations of background-adapted threshold- and fixed threshold-based segmentation. Overall, the use of increasing relative thresholds resulted in decreasing MTVs while higher MTV deviations were observed for smaller spheres. At SBR1 and SBR2, PSF as well as PSF + TOF led to substantial underestimation by all segmentation methods compared to 3D-OSEM, 3D-OSEM + TOF, FBP, and FBP + TOF with lowest mean MTV deviations for t40. At SBR3, only small inter-method differences concerning reconstruction were observed. t40 was not applicable whereas t50 provided the lowest mean MTV deviations for PSF and PSF + TOF. Please see Table 4 for all results.

**Table 4:** **MTV deviations from reference volume**

	ΔMTV (%) [tBC]	ΔMTV (%) [t40]	ΔMTV (%) [t50]	ΔMTV (%) [t60]
**SBR1**				
FBP	-0.5 (-5.7 to 3.3)	3.3 (2.3 to 5.5)*	-8.0 (-13.4 to -5.7)*	-20.0 (-29.5 to -13.3)*
FBP + TOF	0.1 (-3.9 to 2.4)	4.1 (3.2 to 5.1)**	-7.1 (-11.6 to -4.4)*	-18.4 (-25.8 to -11.7)**
3D-OSEM	-4.2 (-8.3 to -2.7)	-1.8 (-3.9 to -1.0)	-10.4 (-14.5 to -7.5)**	-20.3 (-28.0 to -13.7)**
3D-OSEM + TOF	-2.6 (-5.4 to -1.3)	0.5 (-1.0 to 2.0)	-8.1 (-11.6 to -5.7)**	-17.6 (-24.4 to -11.8)**
PSF	-9.4 (-15.2 to -5.5)*	-8.1 (-14.1 to -4.0)*	-15.8 (-22.5 to -9.7)*	-24.6 (-35.7 to -14.9)*
PSF + TOF	-9.2 (-16.7 to -4.9)*	-7.3 (-14.1 to -3.7)	-15.2 (-24.7 to -9.2)*	-23.0 (-35.3 to -14.2)*
**SBR2**				
FBP	-3.2 (-5.7 to -2.2)*	9.7 (5.2 to 15.8)*	-5.0 (-6.8 to -3.8)**	-17.9 (-23.6 to -12.2)**
FBP + TOF	-2.1 (-4.6 to -0.7)	10.6 (6.3 to 15.8)*	-3.7 (-5.0 to -2.6)**	-17.0 (-23.6 to -10.7)**
3D-OSEM	-8.2 (-12.7 to -4.7)*	2.0 (-0.3 to 5.2)	-11.3 (-16.0 to -6.4)*	-21.7 (-28.0 to -13.9)**
3D-OSEM + TOF	-5.9 (-10.9 to -3.3)*	4.9 (3.2 to 7.4)*	-8.3 (-12.7 to -4.5)*	-18.8 (-25.1 to -11.7)**
PSF	-11.7 (-18.9 to -5.9)*	-2.1 (-4.6 to 0.0)	-14.5 (-21.1 to -7.4)*	-24.0 (-33.5 to -14.1)*
PSF + TOF	-10.5 (-18.9 to -5.3)*	-1.4 (-4.3 to 0.6)	-13.1 (-21.1 to -7.2)*	-22.7 (-33.1 to -14.0)*
**SBR3**				
FBP	-17.5 (-26.2 to -13.2)**	Segmentation not applicable	12.4 (-1.0 to 24.2)	-15.4 (-21.5 to -11.2)**
FBP + TOF	-14.5 (-22.5 to -11.5)*		14.8 (3.0 to 26.1)	-11.2 (-16.3 to -8.3)**
3D-OSEM	-17.2 (-28.0 to -12.7)*		6.3 (1.7 to 9.1)*	-15.0 (-23.6 to -10.3)*
3D-OSEM + TOF	-14.4 (-24.7 to -10.1)*		10.6 (4.0 to 14.7)*	-11.6 (-20.7 to -7.5)*
PSF	-16.3 (-23.6 to -11.2)**		9.6 (2.5 to 17.7)	-13.1 (-17.4 to -9.4)**
PSF + TOF	-16.3 (-26.2 to -11.3)*		5.3 (0.8 to 13.0)	-14.3 (-24.4 to -7.5)*

Mean (range) relative deviations from the reference volume of all spheres are displayed for each segmentation method, SBR, and reconstruction method. *t* test: *P < 0.05; **P < 0.01; ***P < 0.001.

The GLM showed a significant association of the relative MTV deviation with reconstruction algorithm (reference method, 3D-OSEM; FBP, *P* = 0.15; FBP + TOF, *P* < 0.05; 3D-OSEM + TOF, *P* = 0.08; PSF, *P* < 0.01; PSF + TOF, *P* < 0.05), sphere diameter (*P* < 0.001), and SBR (reference SBR, SBR1; SBR2, *P* < 0.05; SBR3, *P* < 0.001) but not with the slice thickness of the reconstructed PET data (*P* = 0.20).

## Discussion

In the present study, phantom measurements were performed to examine the influence of different reconstruction algorithms and SBRs on quantitative FDG-PET. We showed that PSF + TOF provided a significantly improved spatial resolution compared to all other investigated reconstruction algorithms but differences are dependent on the SBR (Figure 2). Also, the investigated OSEM reconstructions showed a SBR-dependent spatial resolution. The reason for this is most likely a contrast-dependent convergence of the iterative reconstructions. This of course suggests optimizing the reconstruction parameters for each contrast, but this would not be possible for clinical data. There, the target structures can feature a wide range of SBRs. An optimization of the parameters for all SBRs at the same time is not possible and, therefore, was not performed for the present phantom data either. Thus, we used the parameters recommended by the manufacturer of the PET/CT scanner for each reconstruction.

In contrast to the present study, the National Electrical Manufacturers Association (NEMA) recommends a standardized phantom architecture including six point sources of less than 1-mm diameter surrounded by air to calculate the spatial resolution from the FWHM of several one-dimensional activity profiles [21]. No scatter medium and no background are present in such measurements. Our approach allows computing the spatial resolution also with extended objects in a finite background, which is much closer to the clinical situation than point sources in air.

The radial activity profiles of the PSF algorithms revealed signal elevation at the boundaries of the spheres. These elevations are known as Gibbs artifacts and have been shown to be intrinsic for PSF reconstruction algorithms [22]. Gibbs artifacts appear near sharp transitions from high to low signal, and the absolute value depends on the height of the signal's jump (SBR) [23] and the level of the resolution recovery. The relative magnitude of these artifacts, however, depends on the resolution recovery only (artifacts get stronger with lower FWHM) - rendering them visible only at SBR1 and SBR2 as can be seen in Figures 3 and 4. Also, the quantitative results for GA (Table 2) directly depend on the contrast. At SBR1, GA for PSF + TOF of the largest sphere (diameter, 70 mm) was 6.3%; at SBR2, GA was 5.0%; and at SBR3, GA was reduced to 2.7%. The results for the 50-mm sphere are similar. The diameter of the two smallest spheres was too small for a detection of local minima (see above), and, therefore, a quantification of the Gibbs artifacts was not possible. However, Figure 4G,H,I clearly shows that Gibbs artifacts are present at high contrast also for these diameters and are essentially absent at low contrast.

The edge elevations result in an artificially increased contrast of hot structures which has been reported to yield improved visual lesion detectability, especially if combined with TOF analysis [24, 25]. However, the current results imply that at low SBR no considerable advantages of PSF can be expected. Thus, the specific influence of different SBRs on the abovementioned effects and, moreover, the role of Gibbs artifacts in clinical practice requires further investigations.

As a direct consequence of these artifacts, both PSF algorithms resulted in a significantly higher deviation of the SUVmax from the reference SUV at SBR1 and SBR2 (up to about 40% for the smallest sphere) compared to 3D-OSEM- and FBP-based data. These results are in agreement with phantom measurements performed by Prieto et al. [10] also using a Siemens Biograph mCT 64 scanner and sphere diameters ranging from 10.1 to 37.6 mm. As at present the SUVmax is the most common quantitative parameter used for outcome prediction, therapy response assessment, and threshold-based target volume definition in oncology [4, 26, 27], these findings are of substantial clinical relevance. The presence of Gibbs artifacts dependent on the contrast can cause additional problems. Consider, for example, the therapy response assessment of liver metastases. The liver typically features an SUV of 2. A metastasis with an SUV of 12 would then correspond to SBR2, and the measured SUV would be overestimated due to Gibbs artifacts. Assuming that during therapy the SUV drops to 4.6, it would then correspond to SBR3. At this contrast, essentially, no Gibbs artifacts are present and, therefore, there is also no overestimation of the measured SUV. In consequence, the response assessment can be affected as the difference of these SUV values is larger than the actual difference.

Compared to SUVmax, the SUVmean showed smaller deviations from the reference SUV for all reconstruction algorithms (lowest for PSF and PSF + TOF). These observations confirm results of recent studies [10, 28]. In the study by Prieto et al. [10], the authors analyzed the influence of different reconstruction methods (FBP, OSEM, PSF, PSF + TOF) on SUVmean within an isocontour of 50% of the SUVmax (SUV50). PSF + TOF provided the lowest relative deviation from the true value (median, 0.3%; *P* = 0.34). The present study revealed comparable results for t50 which showed the lowest deviation from the reference SUV for PSF + TOF (mean, -2.6%; *P* = 0.14).

For volume delineation, we used an adaptive threshold method and three different fixed thresholds for comparison. At high and medium contrasts, adaptive as well as fixed thresholding of PSF- and PSF + TOF-reconstructed images resulted in significantly higher MTV deviations from the reference volume compared to FBP-based or 3D-OSEM-reconstructed images. However, the deviations were rather small. Only for the smallest sphere the deviation exceeded 18% (delineated with tBC) compared to 13% with 3D-OSEM and 6% with FBP.

Knäusl et al. delineated distinctly smaller target volumes (0.3 to 11.5 ml) and reported MTV underestimation up to 39% using PSF compared to OSEM [12]. For the smallest sphere investigated in the present study (14 ml), t40 delineation resulted in a difference between PSF and OSEM of only 9%. However, an extrapolation of the data in Figure 6 (t40, SBR1) to smaller volumes would result in a similar difference between PSF and OSEM as reported in [12].

In a further study, Knäusl et al. observed lower relative thresholds delineating the true sphere volume for PSF compared to OSEM which is in accordance with the observed MTV underestimation in the present study. The authors reported further that threshold differences between PSF and OSEM increased with increasing SBR [11] corresponding to larger differences in relative MTV deviations between PSF + TOF and 3D-OSEM at higher SBR. This is confirmed by the present study revealing that the optimal fixed threshold depends on both the reconstruction method as well as the SBR (Figure 6). Knäusl et al. showed that MTV deviations caused by increased SUVmax in PSF-reconstructed data can be minimized by calibrating the volume reproducing threshold for these reconstruction algorithms separately [12]. However, this approach was only applied to lung lesions (with typically high tumor-to-background ratios). As the current results underline that the PSF-related MTV deviations must be assessed considering the respective SBR, it remains questionable whether this approach could be an adequate and feasible method under clinical conditions.

A limitation of the present study is that spherical inserts with cold walls were used. The cold walls introduce a delineation error for threshold-based delineation methods which depends on the size of the walls, the spatial resolution, and the contrast [17]. However, at high contrast, these delineation errors are very small. Therefore, the result that both PSF reconstructions lead to an underestimated volume at high contrast, when delineated with threshold-based methods, is not affected by the cold walls of the spheres. The situation is different at low contrast (SBR3). First, there is no notable difference in tBC delineation between PSF algorithms and the other investigated reconstruction algorithms, which is explained by the absence of Gibbs artifacts at this contrast. Second, for all reconstruction algorithms, tBC delineation underestimated the actual volumes of all investigated spheres. This is mainly caused by the effects of the cold walls. At low contrast, the cold walls lead to an underestimated volume when a threshold is used which was optimized for data without walls, e.g., clinical data. The optimization of this algorithm for such spheres would require a calibration which takes the cold walls into account. However, such a calibration would be only of limited use since it is only valid for the type of spheres the calibration was performed with. An alternative would be the use of spheres without cold walls as performed by Bazañez-Borgert et al. [29]. However, the main result at low contrast, namely that there is no difference in tBC delineation between PSF-based and other reconstructions, could also be shown with the presented measurements.

Another limitation in the same context is that only threshold-based delineation methods were used. Other non-threshold-based methods (e.g., [30]–[35]) might perform better with PSF-reconstructed data. Such methods are not available at our site and could not be investigated. Therefore, the reported MTV deviations of PSF reconstructions are strictly speaking only valid for threshold-based delineation methods.

## Conclusions

At high contrast, the PSF algorithms provided the highest spatial resolution and lowest SUVmean deviation from the reference SUV. In contrast, both algorithms showed the highest deviations in SUVmax and threshold-based MTV definition. At low contrast, all investigated reconstruction algorithms performed approximately equally. The use of PSF algorithms for quantitative PET data, e.g., for target volume definition or in serial PET studies, should be performed with caution - especially if SUV of lesions with high and low contrasts are compared.

## Authors’ original submitted files for images

Below are the links to the authors’ original submitted files for images. Authors’ original file for figure 1
Authors’ original file for figure 2
Authors’ original file for figure 3
Authors’ original file for figure 4
Authors’ original file for figure 5
Authors’ original file for figure 6
