# Supplementary figures and images for: The influence of different signal-to-background ratios on spatial resolution and F18-FDG-PET quantification using point spread function and time-of-flight reconstruction
Source: EJNMMI Phys. 2014 Sep 19;1:12. doi: 10.1186/2197-7364-1-12 (PMC6890905; doi:10.1186/2197-7364-1-12)

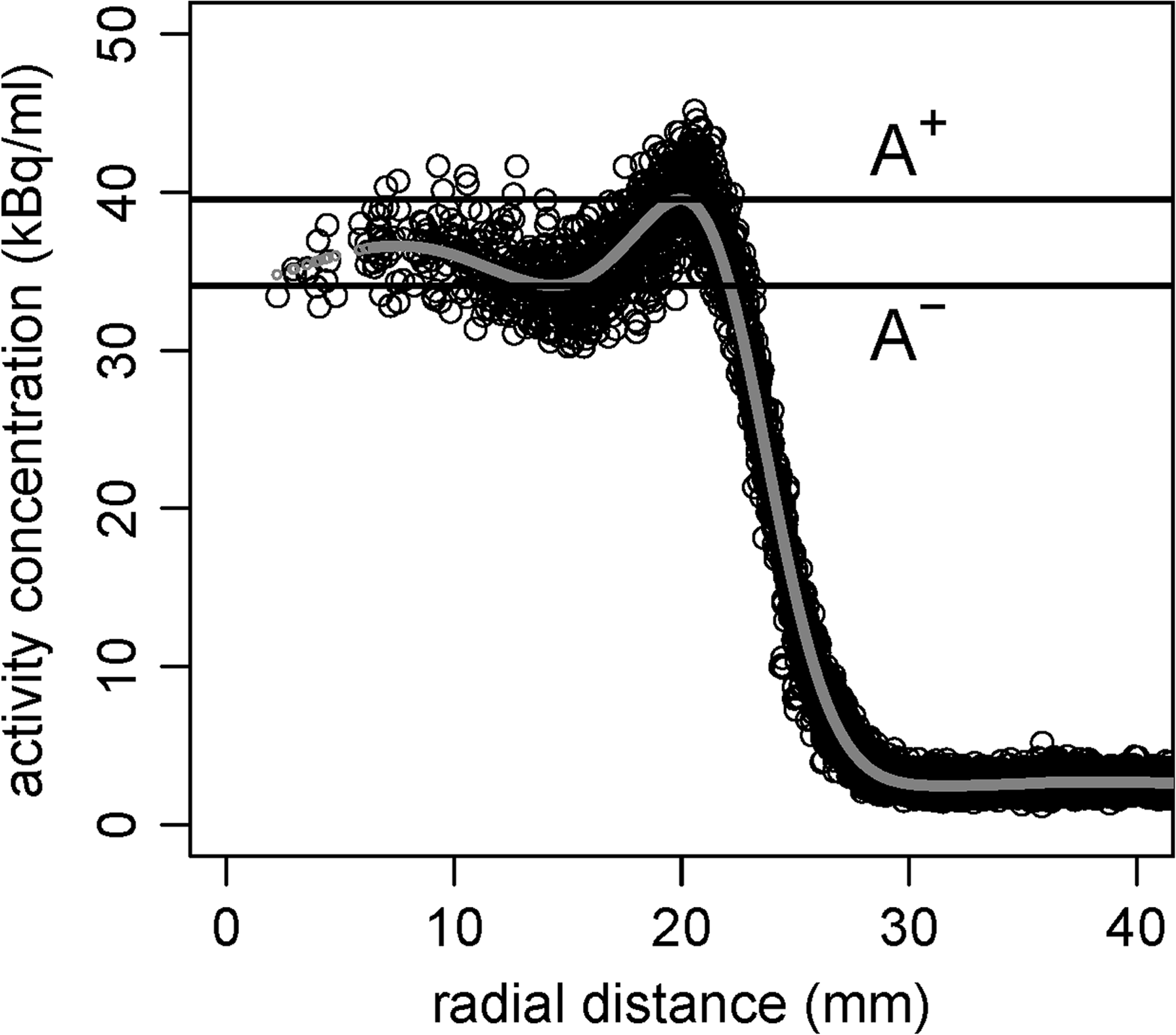

Supplement: Supplementary file 1 — Authors’ original file for figure 1 [file 40658_2014_9003_MOESM1_ESM.tiff]

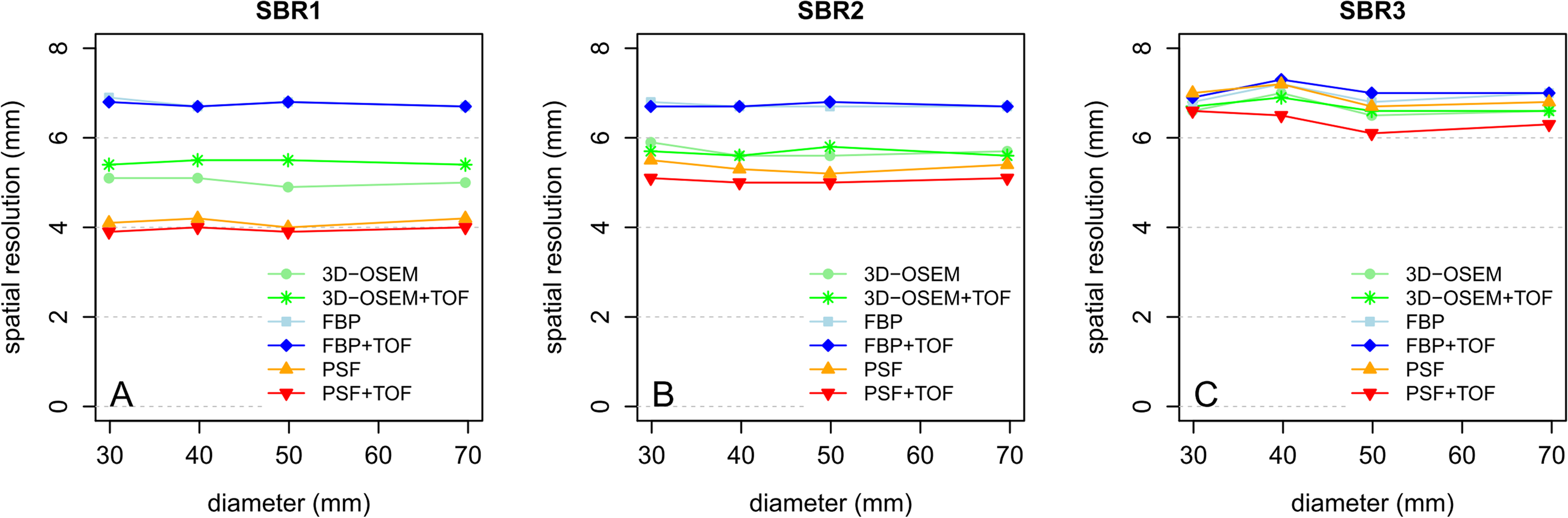

Supplement: Supplementary file 2 — Authors’ original file for figure 2 [file 40658_2014_9003_MOESM2_ESM.tiff]

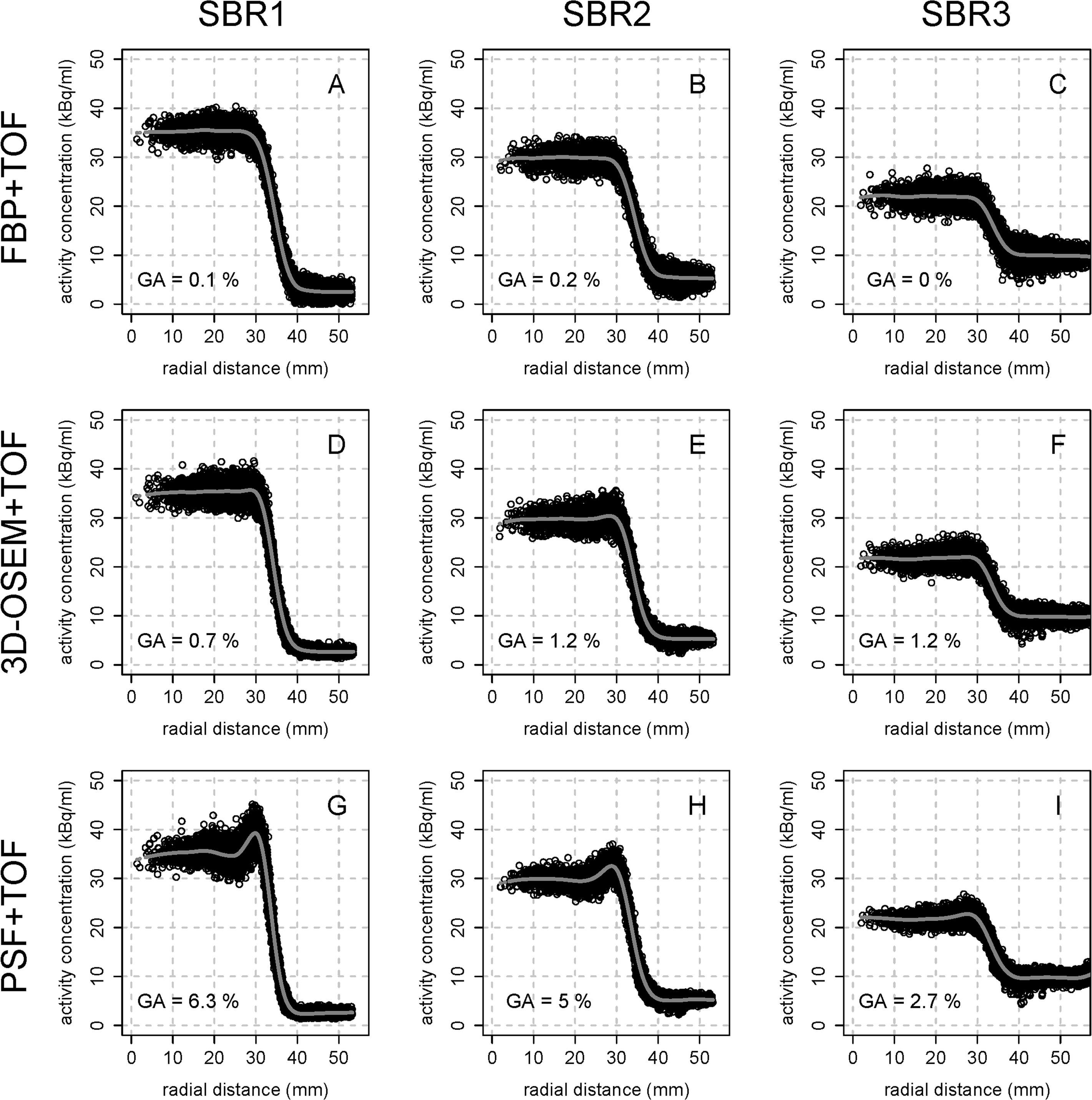

Supplement: Supplementary file 3 — Authors’ original file for figure 3 [file 40658_2014_9003_MOESM3_ESM.tiff]

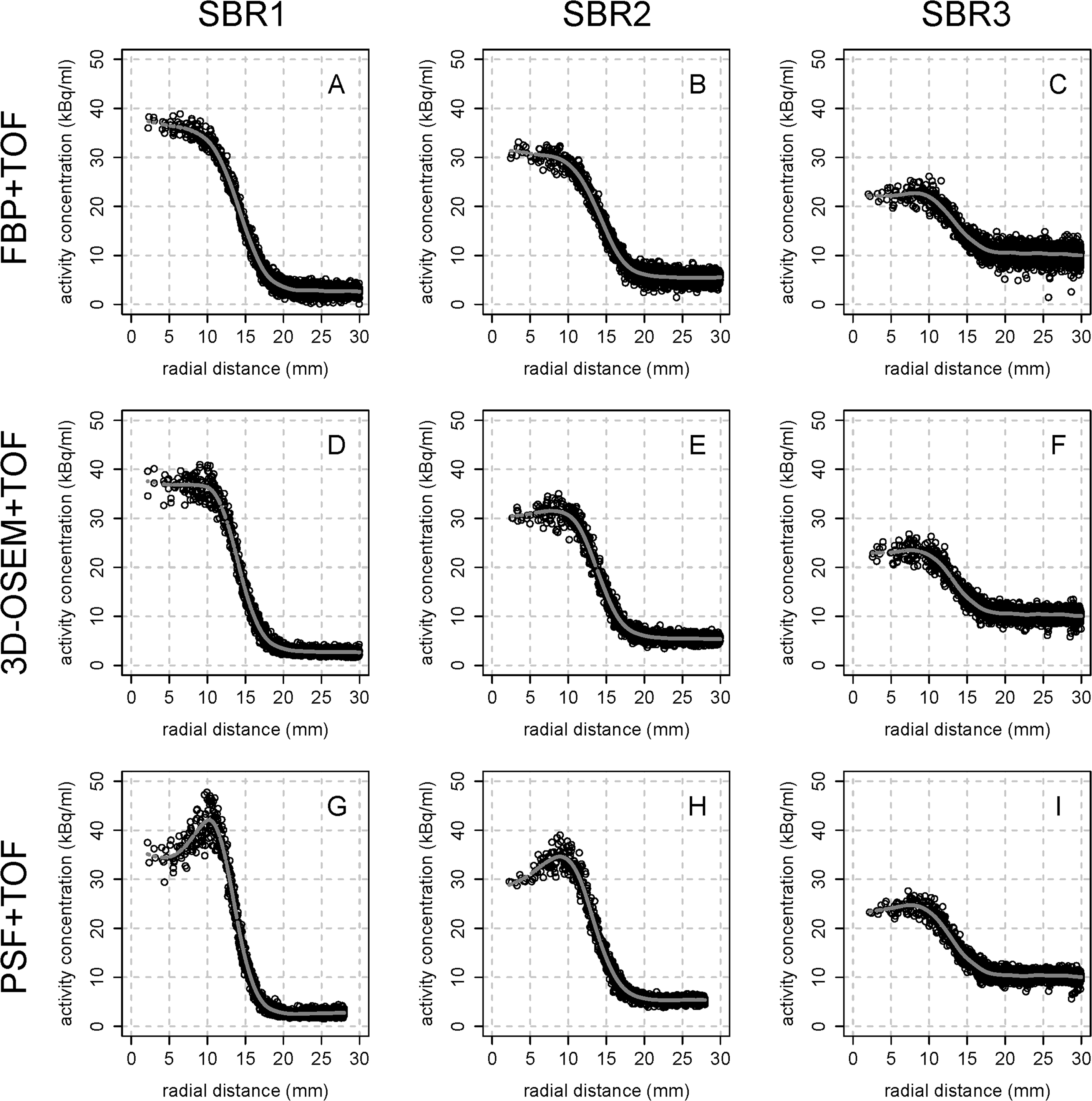

Supplement: Supplementary file 4 — Authors’ original file for figure 4 [file 40658_2014_9003_MOESM4_ESM.tiff]

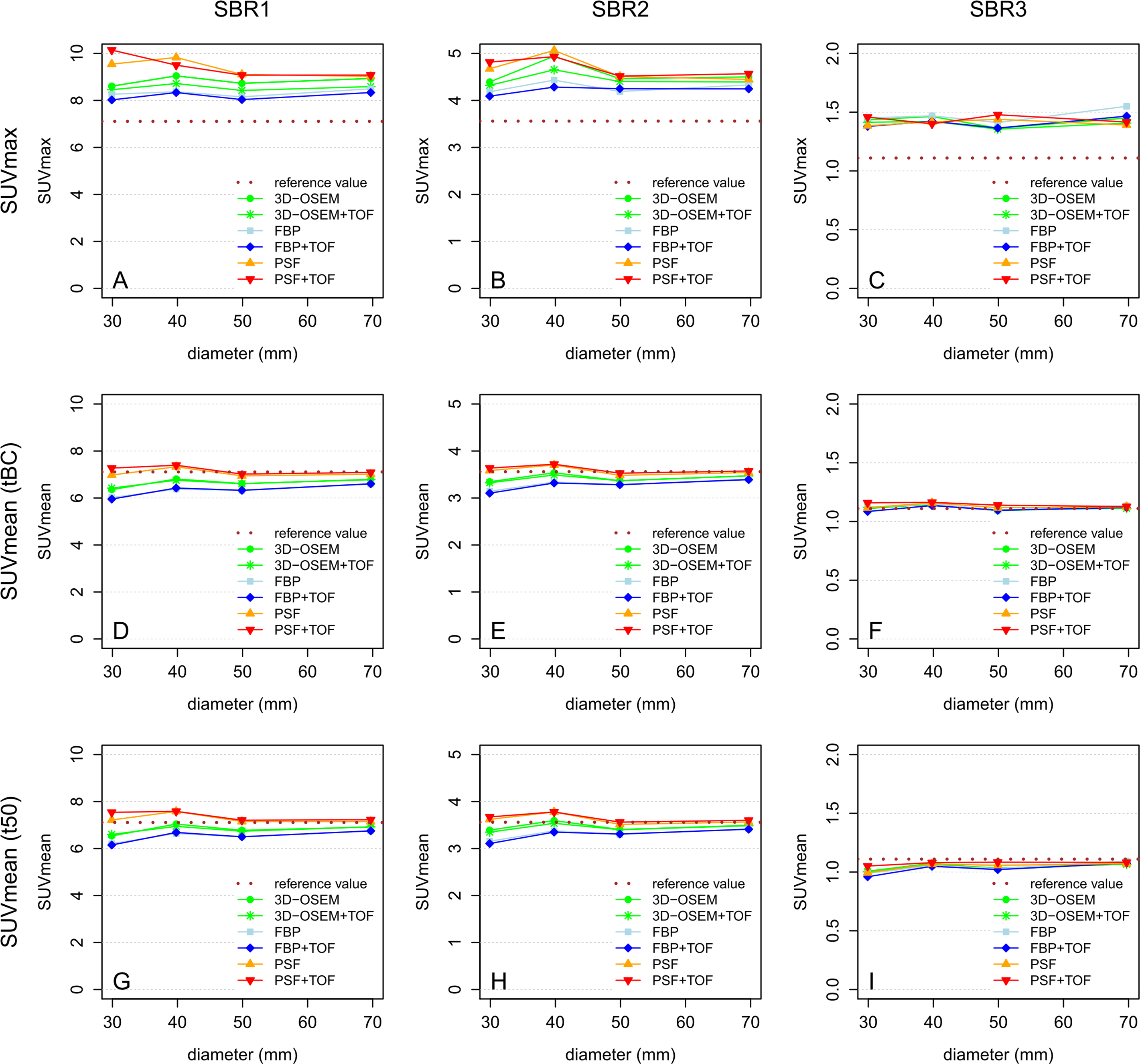

Supplement: Supplementary file 5 — Authors’ original file for figure 5 [file 40658_2014_9003_MOESM5_ESM.tiff]

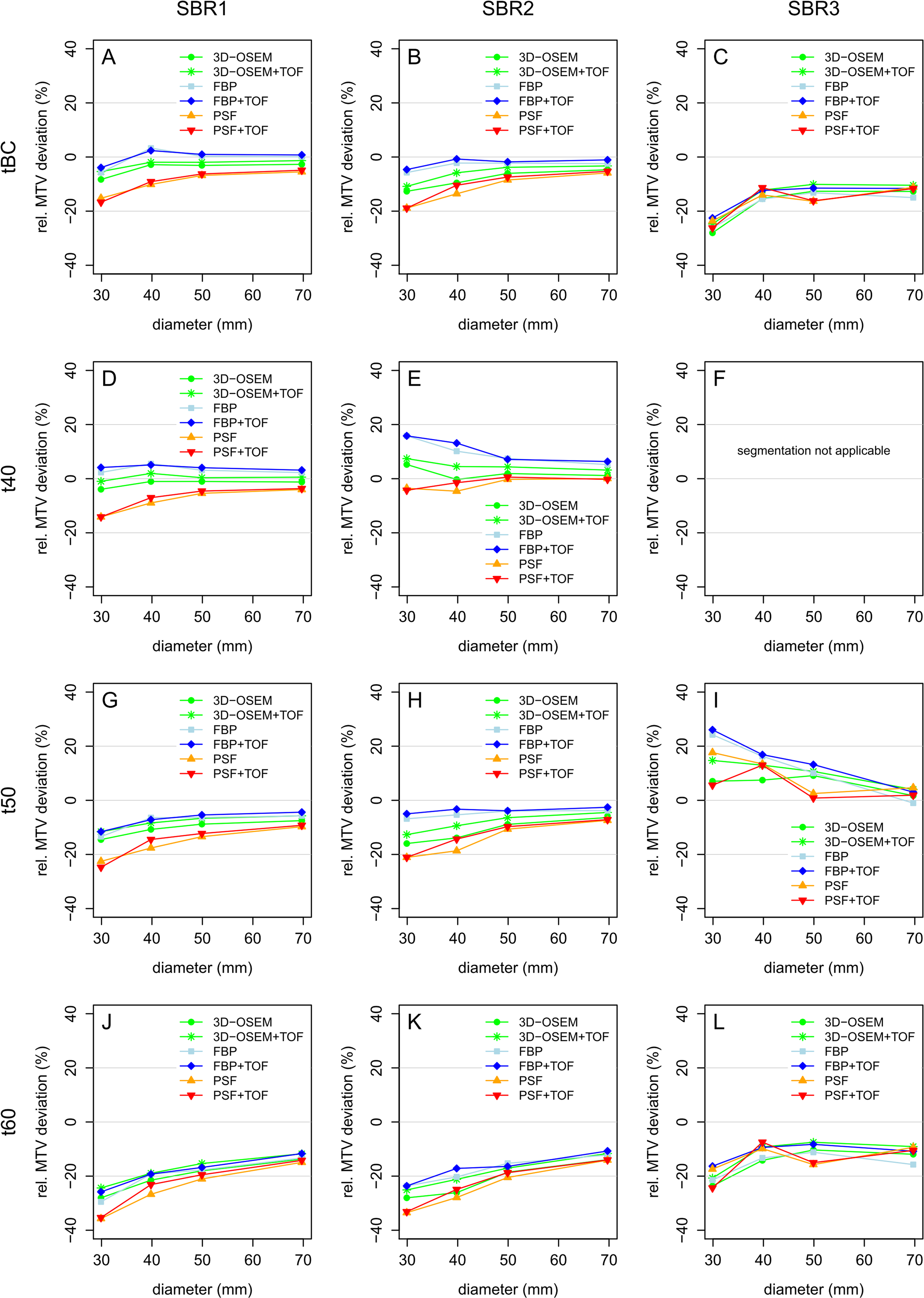

Supplement: Supplementary file 6 — Authors’ original file for figure 6 [file 40658_2014_9003_MOESM6_ESM.tiff]
